# Paediatric colistin prescribing practices in South Africa: A clinician survey

**DOI:** 10.4102/sajid.v40i1.730

**Published:** 2025-08-05

**Authors:** Veshni Pillay-Fuentes Lorente, Trusha Nana, Marianne Black, Gary Reubenson, Reenu Thomas, Angela Dramowski, Adrie Bekker, Nicolette du Plessis, Despina Demopoulos, Vindana Chibabhai

**Affiliations:** 1 Division of Clinical Pharmacology, Department of Medicine, Faculty of Medicine and Health Sciences, Stellenbosch University, Cape Town, South Africa; 2 Division of Clinical Microbiology and Infectious Diseases, School of Pathology, Faculty of Health Sciences, University of the Witwatersrand, Johannesburg, South Africa; 3 National Health Laboratory Service, Johannesburg, South Africa; 4 Lancet Laboratories, Johannesburg, South Africa; 5 Department of Paediatrics and Child Health, Faculty of Health Sciences, University of the Witwatersrand, Johannesburg, South Africa; 6 Division of Neonatology, Department of Paediatrics, Faculty of Health Sciences, University of the Witwatersrand, Johannesburg, South Africa; 7 Department of Paediatrics and Child Health, Stellenbosch University, Cape Town, South Africa; 8 Division of Paediatric Infectious Diseases, Department of Paediatrics, University of Pretoria, Tshwane, South Africa; 9 Centre for Healthcare Associated Infections, Antimicrobial Resistance and Mycoses, National Institute for Communicable Diseases of the National Health Laboratory Service, Johannesburg, South Africa

**Keywords:** colistimethate sodium, polymixin, antimicrobial stewardship, colistin, multi-drug resistant gram negative bacteria

## Abstract

**Background:**

Increasing multidrug-resistant bacterial infections are a global health challenge. Colistin, a polymyxin antimicrobial, has activity against some resistant strains, and despite its adverse effects, it presents a last-line option to treat resistant Gram-negative pathogens. However, paediatric colistin prescribing guidelines are lacking.

**Objectives:**

To determine paediatric colistin prescribing practices and challenges in South Africa (SA) to aid the development of a paediatric colistin guideline.

**Method:**

We conducted an anonymous online survey of registered medical practitioners in SA who prescribed colistin to patients aged ≤ 14 years in the past 12 months.

**Results:**

Of 196 participants, 71.9% (*n* = 141/196) completed the survey. Eighty-six respondents (*n* = 86/146; 58.9%) reported prescribing loading doses (LD), with the median LD and maintenance doses of 150 000 IU/kg/dose (interquartile range (IQR), 75 000–150 000) and 50 000 IU/kg/dose (IQR, 40 000–50 000), respectively. Empiric colistin use was reported by 47.2% (*n* = 69/146), of whom 46.3% (*n* = 32/69) continued empiric colistin for ≥ 72 h. Using the Likert scale, respondents highly perceived that therapeutic drug monitoring should be readily available (mean = 3.97). The perception that prescribing colistin should be advised by a microbiologist or infectious disease specialist had a mean score of 2.97, indicating moderate agreement.

**Conclusion:**

This survey demonstrated varied paediatric colistin prescribing practices. Recently, a new evidence-based paediatric guideline for colistin use in SA has been published. A follow-up survey will be conducted to assess the impact of the guideline on paediatric colistin prescribing practices in SA.

**Contribution:**

This study highlighted paediatric colistin dosing practices in the absence of a paediatric colistin guideline in South Africa.

## Introduction

Antimicrobial resistance (AMR) has been identified as a health challenge, which can be addressed through the One Health approach.^1^ *One Health* is an integrated approach to improve health by taking into consideration that the health of ecosystems and those of humans and animals are linked and interdependent.^1^ If no action is taken to address AMR, it could contribute annually to 10 million deaths by 2050.^2^ In 2019, 1.2 million deaths resulted as a direct consequence of AMR.^3^ To address this challenge of AMR, the World Health Organization (WHO) updated the 2017 edition of the ‘WHO Bacterial Priority Pathogens List’ in 2024 to classify pathogens based on priority to guide research and the development of health interventions.^4^ Priority pathogens are categorised as critical-group, high-group or medium-group pathogens.^4^ Critical-group pathogens are a high public health threat because of their limited treatment options. These pathogens also possess a high morbidity and mortality. High-group pathogens are difficult to treat, are highly transmissible and have demonstrated increasing resistance. Medium-group pathogens are moderately difficult to treat with a moderate increase in resistance. The pathogens categorised as critical priority are all Gram-negative bacteria and include third-generation cephalosporin-resistant Enterobacterales (3GCRE), carbapenem-resistant Enterobacterales (CRE) and carbapenem-resistant *Acinetobacter baumannii* (CRAB).^4^ Carbapenem-resistant *Pseudomonas aeruginosa* (CRPsA) is regarded as a high-priority pathogen.^4^

Resource constraints in low- and middle-income countries (LMICs) make newer or more expensive antimicrobials such as tigecycline, ceftazidime-avibactam, cefiderocol and sulbactam-durlobactam difficult to obtain. Hence, in these settings, colistin, a polymyxin antimicrobial agent targeting the bacterial cell membrane,^5^ remains the last-line antibiotic to treat multidrug-resistant Gram-negative bacteria (MDR-GNB). In South Africa, colistin may only be prescribed under a Section 21 application by the medical practitioner and subsequent approval by the South African Health Regulatory Authority (SAHPRA).^6^ Section 21 applications allow for colistin, which is an unregistered medicine in South Africa, to be administered in instances where conventional therapies have failed or are unavailable; however, the application requires patient or caregiver consent. This extra administrative work related to the application process and delays in approval by SAHPRA may hinder the prompt initiation of this antibiotic.

Despite the reduced use of colistin in the clinical setting in the late 1960s due to its reported adverse effects, colistin has recently regained favour among clinicians due to the surge of MDR-GNB infections globally, where colistin is reported to be acceptably effective.^7^ In the early stages of drug development, microbiological studies rather than clinical trials supported the efficacy and dosing of colistin.^7^ Subsequently, clinical studies in adults have contributed to a well-developed adult colistin guideline, which has been endorsed by various societies.^8^ However, prior to the interim guidance on appropriate colistin use published recently (2023),^9^ there was a paucity of locally and internationally endorsed consensus guidelines for colistin prescribing in paediatrics, with the last local dosing recommendations for colistin in the paediatric population published in 2016.^10^ With the increasing use of colistin, there was a need for paediatric guidelines when prescribing colistin to adequately preserve its effectiveness and optimise patient outcomes.

A group of paediatricians comprising infectious disease specialists, microbiologists, neonatologists and a clinical pharmacologist sought to develop a paediatric colistin prescribing guideline. The objective of this study, prior to the availability of the abovementioned guideline, was to understand and describe colistin prescribing practices and attitudes and the barriers to prescribing colistin for neonates, infants and children in SA. As prescribing of medication in most of the South African public sector hospitals is paper based, with no current centralised system to monitor prescribing practices, a survey was considered a reasonable method by which to determine the prescribing practices of colistin as a baseline.

## Research methods and design

### Study design and eligibility

An anonymous, cross-sectional, web-based survey was conducted between 08 June 2023 and 18 July 2023. The survey was conducted in English. Registered medical practitioners practising in SA were eligible to participate in the survey if they had prescribed colistin to a neonate, infant or child aged ≤ 14 years in the preceding 12 months.

### Survey distribution

Distribution of the survey was achieved through Essential Medicine Guidance (EMGuidance) (https://info.emguidance.com/), a free, mobile and web-based digital platform and through three South African clinical organisations: the Federation of Infectious Diseases Societies of Southern Africa (FIDSSA), the South African Paediatric Association (SAPA) and the United South African Neonatal Association (USANA). These organisations were selected because their members include a considerable number of South African paediatricians. At the time of conducting this study, FIDSSA had 92 members who were part of the South African Society of Paediatric Infectious Diseases, SAPA had a total of 2362 members and USANA had 189 members. South African Paediatric Association members included paediatric specialists as well as allied professionals caring for children. An email invitation to voluntarily complete the survey was circulated to the respective organisations. Essential Medicine Guidance is an independent platform available to all healthcare professionals and is often utilised by clinicians in SA. A link and Quick Response (QR) code to the survey were posted on the home page of EMGuidance for the duration of the study.

### Data collection

The survey was hosted on the Research Electronic Data Capture (REDCap, https://projectredcap.org/software/) database at Stellenbosch University, Cape Town, SA. Clinicians were asked questions pertaining to prescriber- and practice-setting characteristics, prescribing practices including loading and maintenance doses, duration of therapy, monitoring adverse events and inflammatory markers, Antimicrobial Stewardship (AMS) committee roles in authorising colistin use and attitudes and barriers to prescribing colistin (Online Appendix, Table 1-A1). Attitudes towards prescribing colistin were assessed using a 5-point Likert scale where strongly disagree = 1, disagree = 2, neutral = 3, agree = 4 and strongly agree = 5. A convenience sampling strategy was used, and no sample size calculation was conducted.

### Statistical analysis

Data were exported from the REDCap database and imported into R (R Core Team, 2024, Vienna, Austria, version 4.4.0) and RStudio (RStudio Team, 2023, Boston, version 2024.04.0+735) for analysis. R packages were used to analyse the data and to construct the graphs. Participant characteristics were described using percentages and frequencies for binary data. Continuous data that were skewed were reported as medians and interquartile ranges. Chi-square tests were used to assess associations between variables of interest. The consistency of the Likert scale was assessed using the Cronbach alpha test, and decisions on perceptions were made using the weighted average of the mean. A non-probability sampling method was used.

### Ethical considerations

Participants were required to consent to participation before completing the survey. Ethical clearance to conduct this study was obtained from the University of the Witwatersrand Human Research Ethics Committee (Medical) (No. M230330).

## Results

A total of 200 participants engaged with the consent page of the survey. Four clinicians declined to participate, and 196 participants consented to take the survey. Of the 196 participants, 71.9% (*n* = 141/196) completed the survey, 2.5% (*n* = 5/196) partially responded and 25.5% (*n* = 50/196) consented to participate in the survey but did not answer any survey questions.

### Hospital setting

Some respondents worked in more than one setting. Of the respondents, 3.4% (*n* = 5/145) worked in a district-level public hospital, 35.8% (*n* = 52/145) in a regional-level public hospital, 65.5% (*n* = 95/145) in a central-public hospital and 15.1% (*n* = 22/145) in a private hospital. The definitions of the public sector hospitals are represented in the Online Appendix, Table 2-A1.

Respondents prescribed colistin more frequently in central hospital settings (*n* = 95/146, 65.1%) and regional hospital settings (*n* = 43/146, 29.4%) than in district-level (*n* = 3/146, 2.1%) and private (*n* = 5/146, 3.4%) settings. Fourteen per cent (*n* = 13/95) of respondents who prescribed colistin in central settings indicated that it was not readily available in their institutions. Of the respondents who prescribed colistin more frequently in regional hospitals, 32.5% (*n* = 14/43) indicated that colistin is not readily available. Of the respondents prescribing colistin in a district-level hospital, 33% (*n* = 1/3) readily acquired colistin. Antibiotics accessible by clinicians in the clinical environments are highlighted in Online Appendix Figure 1-A1.

### Colistin dosing practices

Eighty-seven per cent (*n* = 127/146) of respondents prescribe colistin in international units per kilogram (IU/kg), and 13.0% (*n* = 19/146) of respondents prescribe in mg colistin base activity per kilogram (mg CBA/kg). Table 1 represents the loading dose and maintenance doses commonly prescribed by the survey respondents (Online Appendix, Figure 2-A1 and Online Appendix, Figure 3-A1). Sixty-six (45.2%, *n* = 66/146) of respondents report always prescribing a colistin loading dose, 13.6% (*n* = 20/146) indicate prescribing a loading dose in some circumstances and 41.1% (*n* = 60/146) never prescribe a colistin loading dose at the initiation of colistin therapy. Using the chi-square test, it was determined that regional or central hospital settings were not associated with administering a loading dose of colistin (chi-square statistic (χ^2^) = 0.08, degrees of freedom (*df*) = 1, *p*-value = 0.8). Among the eighty-six respondents who prescribe LD, 75.5% (*n* = 65/86) prescribe the first maintenance dose 8 hours after the loading dose, 18.6% (*n* = 16/86) after 12 h and 5.8% (*n* = 5/86) after 24 h. Ninety-four per cent (*n* = 61/65) of respondents who prescribed the first maintenance dose 8 h after the loading dose prescribed the maintenance doses at 8-h intervals. Among respondents who prescribed the first maintenance dose after 12 h, 93.7% (*n* = 15/16) prescribed the subsequent maintenance doses at 12-h intervals. Sixty per cent (*n* = 3/5) of respondents who administered the first maintenance dose 24 h after the loading dose prescribed subsequent maintenance doses at 8-h intervals, and 40% (*n* = 2/5) used 12-h intervals. Among respondents, colistin is commonly prescribed at 8-h dosing intervals (*n* = 113/146, 77.3%), followed by 12-h (*n* = 30/146, 20.5%) and 24-h (*n* = 3/146, 2.1%) intervals.

**TABLE 1:** Paediatric colistin loading doses and maintenance doses used by respondents.

Dose	Sample†		Median	IQR	Min-max
	*n*	%			
**Loading dose (mg)†**					
CBA/kg/dose	6/86	7.0	3.60	2.68–4.75	2–5
IU/kg/dose	77/86	89.5	150 000	75 000–150 000	0–1 500 000
**Maintenance dose (mg)**					
CBA/kg/dose	16/146	11.0	2.5	2–5	2–10
IU/kg/dose	122/146	83.6	50 000	40 000–50 000	250–3 000 000

Note: IQR = 25th–75th percentile.

CBA, colistin base activity; IU, International units; IQR, interquartile range; Min, minimum; Max, maximum.

† , 86 respondents reported prescribing a loading dose.

### Colistin administration practices

Most respondents prescribe the loading dose as a bolus infusion (*n* = 51/86, 59.3%), as opposed to an infusion ≥ 30 min (*n* = 35/86, 40.7%). Nearly 25% of respondents (24.6%, *n* = 36/146) report the first administration of colistin within 1 h of prescribing the drug, 52.7% (*n* = 77/146) report administrations between 1 and 6 h after prescribing and 7.5% (*n* = 11/146) report administration within 6 h – 12 h after prescribing. Thirty-one respondents (21.2%, *n* = 31/146) have prescribed colistin through routes other than intravenously. These routes included intrathecal (*n* = 9/31, 29.0%), nebulisation (*n* = 25/31, 80.6%), oral and intramuscular administration (*n* = 2/31, 6.5%). Twenty-three per cent of respondents (*n* = 34/146) report that the prescribing of colistin is limited to the intensive care unit setting.

### Colistin monitoring

One hundred and nine (74.6%, *n* = 109/146) respondents indicated that AMS committees within their institutions monitor colistin prescriptions. Thirty-seven (25.3%, *n* = 37/146) respondents reported that either the AMS committees did not monitor the colistin prescriptions, they were unsure of monitoring by an AMS committee, or an AMS committee did not exist within their institution.

### Cultures and infective markers

The respondents indicated that after prescribing colistin, white cell count (*n* = 129/145, 88.9%) and C-reactive protein (*n* = 129/145, 88.9%) were commonly measured. Seventy per cent of respondents (*n* = 102/145) measured neutrophil counts, and 47.5% (*n* = 69/145) measured procalcitonin. All respondents reported performing blood cultures for patients prescribed colistin (*n* = 145/145, 100%). Ninety-eight respondents (67.5%, *n* = 98/145) performed urine cultures, 75.1% (*n* = 109/145) performed cerebrospinal fluid cultures, 54.4% (*n* = 79/145) performed tracheal aspirate cultures and 22% (*n* = 32/145) performed pus swab cultures. Other samples submitted for culture by 7.6% (*n* = 11/145) of respondents included pleural fluid, sputum (in cystic fibrosis patients) and ascitic or peritoneal fluid.

### Monitoring for side effects

During colistin therapy, respondents reported monitoring for acute kidney injury (*n* = 134/146, 91.7%), neurotoxicity (*n* = 42/146, 28.7%) and electrolyte abnormalities (*n* = 117/146, 80.1%).

### Colistin indications

Respondents reported that the hospital-acquired organisms commonly isolated in the last 12 months in their institutions were extended-spectrum-beta-lactamase (ESBL)-producing Enterobacterales (*n* = 121/144, 84.0%), extensively drug-resistant (XDR) *A. baumannii* (*n* = 114/144, 79.2%), CRE (*n* = 110/144, 76.4%), XDR *P. aeruginosa* (*n* = 23/144, 16.0%), methicillin-resistant *Staphylococcus aureus* (MRSA) (*n* = 87/144, 60.4%) and vancomycin-resistant enterococci (VRE) (*n* = 9/144, 6.3%).

Colistin is prescribed without confirmatory microbiological isolation of a relevant MDR-GNB by 47.2% (*n* = 69/146) of respondents. The majority of respondents reported that empiric therapy is typically prescribed for less than 72 h (*n* = 37/69, 53.6%), with 4.3% (*n* = 3/69) of respondents prescribing empiric colistin therapy for longer than 7 days. Seventy-four per cent (*n* = 108/146) of respondents prescribed colistin in combination with another antimicrobial when treating CRE, and 73.3% (*n* = 107/146) prescribed it when treating XDR *A. baumannii*. Seventy-four respondents (51.4%, *n* = 74/144) used combination therapy with colistin for XDR *P. aeruginosa* infections. Using the chi-square test, it was determined that combination antimicrobial therapy versus monotherapy was not associated with a specific type of resistant organism i.e. XDR *P. aeruginosa*, XDR *A. baumannii* or CRE (χ^2^ = 1.4, *df* = 2, *p*-value = 0.4952). The colistin minimum inhibitory concentrations (MIC) are checked by 62.3% (*n* = 91/146) of respondents when colistin is initiated in response to a positive culture with susceptibility testing available.

### Duration of therapy

**FIGURE 1:**
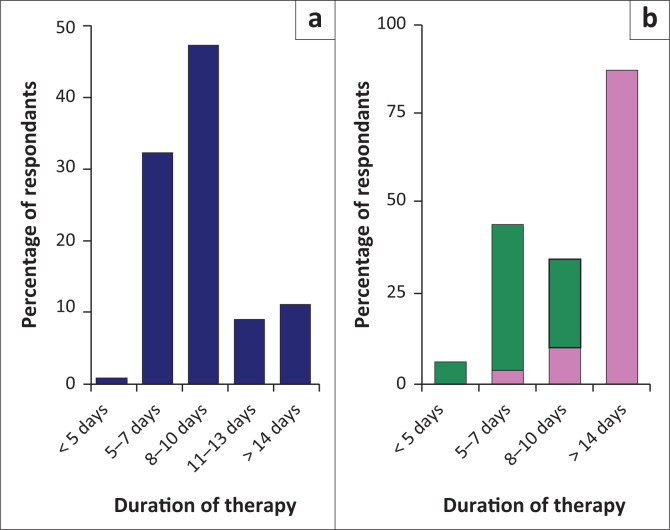
Duration of colistin therapy in colistin-sensitive organisms. (a) Representation of the duration of therapy with blood culture positive for a colistin-sensitive organism. (b) Representation of the duration of therapy for ventilator-associated pneumonia (green) and meningitis (pink) with culture positive for a colistin-sensitive organism.

The durations of colistin therapy for patients with positive blood cultures, ventilator-associated pneumonia (VAP) and meningitis, for colistin-susceptible MDR-GNB or XDR organisms, are shown in Figure 1.

### Perceptions and insights

Table 2 is a representation of respondents’ perceptions of prescribing colistin, measured using the Likert scale described in the Methods section. Most respondents were in agreement that therapeutic drug monitoring should be readily available. This was reflected by a high mean score of 3.97 (Table 2). In contrast, the perception that colistin prescribing should be advised by a microbiologist or infectious disease specialist had a mean score of 2.97, which is slightly above the overall weighted mean of 2.5, indicating moderate agreement. Table 3 provides a summary of the insights gained from the survey questions.

**TABLE 2:** Likert scale assessing attitudes towards colistin prescribing practices among respondents (***N*** = 145).

Items	Strongly disagree		Disagree		Neutral		Agree		Strongly agree		Mean	s.d.	Decision
	*n*	%	*n*	%	*n*	%	*n*	%	*n*	%			
There is little experience prescribing colistin at my institution	46	32	55	38	18	12	20	14	6	4	2.21	1.15	Low perception
Prescribing colistin is avoided because of the lack of adequate dosing guidelines	64	44	64	44	8	6	6	4	3	2	1.76	0.89	Low perception
Prescribing colistin is avoided because of the inconvenience of a Section 21 application	59	40	48	33	16	11	11	8	11	8	2.08	1.23	Low perception
Prescribing colistin is avoided because of its associated adverse effects	54	37	59	41	11	8	15	10	6	4	2.03	1.11	Low perception
Therapeutic drug monitoring of colistin should be readily available	7	5	3	2	21	14	71	49	43	30	3.97	0.98	High perception
Colistin is only prescribed if advised by a microbiologist or an infectious diseases specialist	24	17	39	27	25	17	32	22	25	17	2.97	1.36	High perception

Note: Decision was based on the average = 2.5 (sum of all means of items/number of items). Items with a mean above 2.5 were interpreted as a tendency towards neutral to positive responses. Strongly disagree = 1, disagree = 2, neutral = 3, agree = 4 and strongly agree = 5.

s.d., standard deviation.

**TABLE 3:** Survey insights and colistin guideline recommendations.

Topic surveyed	Observations from survey responses or key variability	Guideline recommendation^9^
Loading dose	- Only 45% reported always prescribing a colistin loading dose at the initiation of therapy. - Only 63% prescribe loading doses within the recommended range. There is a large variability in loading doses prescribed.	All neonates, infants and children requiring colistin should receive a colistin LD of 4 mg – 5 mg colistin base activity (CBA)/kg body weight (118 000 IU/kg–150 150 IU/kg).
Maintenance dose	- Only 8% of individuals prescribed adequate maintenance doses at 12-h intervals. Maintenance dosing regimens varied among respondents.	Maintenance doses of colistin 2.5 mg CBA/kg/dose 12-hourly in children, infants and neonates (74 000 IU/kg).
Colistin administration	- 41% of respondents prescribed the colistin loading dose as an infusion > 30 min.	Colistin may be administered as a slow bolus injection or as a slow infusion over 30 min.
Duration of treatment	- 50% of respondents typically treated a ventilator-associated pneumonia for longer than a week. - 50% of respondents treated meningitis for < 21 days. - Majority of respondents (67%) treated bacteraemia for > 8 days.	Meningitis: Gram-negative meningitis typically treated for 21 days. VAP: typically treated for 5–7 days. Bacteraemia: typically treated for 7 days. (Consult microbiologist/neonatologist/infectious diseases specialist if inadequate clinical response, and considering prolongation of colistin therapy).
Monotherapy or combination therapy	- Approximately 20% of respondents used colistin as monotherapy.	Carbapenemase-producing Enterobacterales (CPE)/CRE: Combination therapy with a second active antibiotic is recommended for severe sepsis/ septic shock. Multidrug-resistant (MDR)/ XDR *Acinetobacter baumannii*: Combination therapy with a second active antibiotic is recommended for severe sepsis or septic shock. If a second active agent is not available, colistin monotherapy is recommended. MDR/XDR *Pseudomonas aeruginosa*: Combination therapy with a second active antibiotic is recommended in those patients with severe sepsis/septic shock.
Adverse events	- Most respondents monitored for acute kidney injury and electrolytes. Only 29% monitored for neurotoxicity.	Monitoring of renal function, sodium, potassium and magnesium, with dosage adjustments when necessary. ICU patients requiring organ support require more frequent monitoring but for patients not requiring organ support, regular monitoring at least once every 72 h. Should be clinician directed. Collect baseline prior to colistin initiation (do not delay initiation/LD while awaiting results). Daily clinical monitoring for neurotoxic side effects is recommended.

Note: Please see full reference list of this article, Lorente VP-F, Nana T, Black M, et al. Paediatric colistin prescribing practices in South Africa: A clinician survey. S Afr J Infect Dis. 2025;40(1), a730. https://doi.org/10.4102/sajid.v40i1.730, for more information.

CRE, carbapenem-resistant Enterobacterales; IU, international units; LD, loading dose; kg, kilogram; ICU, intensive care unit; XDR, extensively drug resistant; VAP, ventilator-associated pneumonia.

## Discussion

A survey was performed to improve the understanding of colistin prescribing practices among clinicians in SA. The survey encouraged stakeholder engagement prior to the development of a consensus guideline for colistin use in paediatrics, which has since been published.^9^ The study found high variability in prescribing practices among clinicians working at different levels of care, warranting a standardised guideline for colistin use in paediatrics.

### AMS committees

The WHO lists colistin as a ‘Reserve’ antibiotic limited to use as a ‘last resort’ in highly specific cases of confirmed or suspected MDR-GNB infections.^10^ ‘Reserve’ antibiotics, such as colistin, should be monitored and preserved through antimicrobial stewardship programmes, ensuring they are used appropriately. In this survey, most respondents prescribed colistin in regional and central hospital settings.^10^ This is likely because there are more cases of MDR-GNB organisms that necessitate the use of ‘Reserve’ antibiotics mostly prescribed by medical specialists. In SA, AMS committees are currently more commonly established within central and regional settings as opposed to district-level hospitals, mainly because of the limited number of available professionals, including the non-availability of microbiologists and infectious disease specialists on site at district health facilities.^11,12,13^ A well-developed colistin guideline could assist facilities without specialist care to use colistin appropriately when its use is indicated. In a recent South African study,^11^ the AMS programmes within academic settings – namely, regional and central hospitals – were less effectively implemented compared to those in non-academic settings (48.9% versus 57.5%), although not statistically significant. In this survey, 74.6% (*n* = 109/146) of respondents who frequently prescribed colistin within regional or central settings reported that AMS teams monitored colistin prescriptions within their environments. On the Likert scale, the statement that ‘colistin is only prescribed if advised by a microbiologist or an infectious diseases specialist’ indicated a strong consensus that colistin access should be controlled by AMS committees. There is a need to strengthen these programmes within the other 25% of hospitals where colistin use is not monitored so that AMS committees can ensure the appropriate use of colistin. In addition, monitoring by national antimicrobial stewardship committees is imperative to ensure countrywide appropriate use of this ‘Reserve’ antibiotic and aligns with the WHO’s strategic action of appropriate use of antimicrobial agents.^3^

### Loading doses

There are two methods of prescribing colistin: either in IU/kg or mg CBA/day. The European Medicines Agency (EMA) recommends 75 000 IU/kg/day – 150 000 IU/kg/day of colistin in children ≤ 40kg, whereas the Food and Drug Administration (FDA) recommends 2.5 mg CBA/kg/day – 5.0 mg CBA/kg/day, equating to 75 000 IU/kg/day – 150 000 IU/kg/day administered intravenously for paediatric patients.^14,15^ In 2016, a South African guideline for colistin use recommended a lower dose for neonates of 50 000 IU/kg/day – 75 000 IU/kg/day.^16^ Subsequent paediatric studies have reported that the recommended FDA and EMA doses were inadequate to achieve acceptable colistin exposures in paediatric patients and that a loading dose may be warranted.^17,18,19^ In 2019, an international consensus guideline on colistin dosing introduced new loading dose recommendations in adult patients. However, this guideline did not include paediatric dosing recommendations.^8^ Current recommended paediatric colistin dosing regimens by the FDA and the EMA do not include a loading dose.^14,15^ Loading doses allow for targeted concentrations to be achieved early in the treatment course.^20^ Furthermore, paediatric patients have a larger body water content, leading to a larger volume of distribution of hydrophilic drugs such as colistin.^21^ A more recent paediatric study reported that administering a loading dose in children achieves better colistin exposure.^18^ Thus, an updated South African guideline informed by published evidence-based studies was needed and has been published.^9^ This study illustrated that 59% of respondents are prescribing LD in paediatrics; however, this seems to be highly variable, with approximately 50% prescribing a loading dose lower than that suggested in published studies.^18^ Maintenance doses per day were mostly prescribed within the recommendations of the regulatory authorities; however, a proportion of respondents prescribed colistin at doses beyond the recommendations enlisted by the FDA and EMA. This could be attributed to studies demonstrating that regulatory dosing recommendations are suboptimal.^17,18,19^ Two respondents reported using high LD of 750 000 IU/kg and 1 500 000 IU/kg, and maintenance doses above the recommended 150 000 IU/kg/day. This could have been erroneously entered by the respondents, but it may represent oversights that could occur in clinical practice when prescribing colistin. Hence, it is imperative that clinicians are trained, and the least complicated dosing strategy is employed (i.e. IU/kg or mg CBA/kg) to reduce prescribing errors. This study reveals that South African respondents prescribe colistin in both IU/kg and in mg CBA/kg. Therefore, guidelines should be provided for both dosing strategies.

The Surviving Sepsis Campaign (SSC) has identified the first hour after the diagnosis of sepsis or septic shock (‘golden hour’) as the most important point to initiate resuscitation and management (including initiating empiric antimicrobials) to improve outcomes.^22^ Notably, only 53% of respondents indicated that colistin initiation was within 1 h of prescribing. This reported practice of delayed antimicrobial administration by 47% of respondents should be addressed by AMS committees considering the clinical environments and resources available, and these committees should implement strategies to aid reduction in the time taken to initiate colistin as this could influence patient outcomes as suggested by the SSC ‘hour-1 bundle’.^22^

### Perceptions

Area under the curve (AUC) and MIC are important metrics when assessing colistin efficacy.^9^ In SA, colistin therapeutic drug monitoring (TDM) is not available, and, in turn, the AUC cannot be calculated for dose optimisation. Seventy-nine per cent (*n* = 114/145) of respondents agreed or strongly agreed with the statement that TDM of colistin should be readily available, suggesting a recognised need for accessible colistin TDM for safe and effective use. Colistin MIC results were not checked by 37.7% (*n* = 55/146) of respondents. Checking the MIC is important to identify whether the bacteria are resistant or susceptible to colistin. According to the Clinical and Laboratory Standards Institute, a MIC of ≤ 2 mg/L is related to intermediate susceptibility.^9^ Furthermore, broth microdilution for colistin susceptibility is performed at central laboratory facilities, and the turnaround time is approximately 1–4 days, which might impact why over one-third of respondents do not check the MIC results. However, AMS committees and guidelines should advocate that clinicians evaluate the reported MIC results to optimise colistin use. High MICs may justify an increase in colistin doses. Respondents did not widely agree that the inconvenience of a Section 21 application through SAHPRA significantly impacted colistin prescribing.

### Adverse effects

Nephrotoxicity and neurotoxicity are reported adverse effects of colistin therapy. This survey demonstrated that renal toxicity is monitored more often than neurotoxicity. Despite neurotoxicity being reported more commonly in older literature compared to newer ones,^23^ all patients on colistin should be monitored routinely for neurotoxicity. However, neurotoxicity may be less observed in children and difficult to recognise.

### Strengths and limitations

This study allowed for stakeholder engagement and provided many insights into colistin prescribing practices, particularly in an LMIC, which is seen as a strength of this study. Notably, this study has limitations. A limitation of this survey includes the small number of respondents despite being widely circulated. Respondents were not asked whether they prescribed for neonates, infants or older children. This could have provided more insight into specific reported dosing and given a better description of dosing challenges within paediatric age groups.

## Conclusion

In conclusion, this study allowed for adequate clinician engagement, which provided insights during the development of a paediatric colistin dosing guideline. This survey demonstrated varied paediatric colistin prescribing and monitoring practices. Furthermore, it highlighted a strong consensus for colistin TDM and the involvement of AMS committees, microbiologists and/or infectious disease specialists in the prescribing of this ‘Reserve‘ antibiotic. Potential barriers to implementation of the guideline include a lack of well-established AMS committees, AMS pharmacists or on-site microbiologists in some hospitals to drive implementation of the guideline through education and training, drug procurement barriers and prolonged laboratory service turnaround times. This survey serves as a clinician prescribing baseline prior to the publication of the paediatric colistin guideline. A follow-up survey is planned to assess the impact and potential barriers to the implementation of the recently published colistin guidance document for paediatrics.
